# Blue light-induced retinal lesions, intraretinal vascular leakage and edema formation in the all-cone mouse retina

**DOI:** 10.1038/cddis.2015.333

**Published:** 2015-11-19

**Authors:** P Geiger, M Barben, C Grimm, M Samardzija

**Affiliations:** 1 Laboratory for Retinal Cell Biology, Department Ophthalmology, USZ, University of Zurich, Switzerland

## Abstract

Little is known about the mechanisms underlying macular degenerations, mainly for the scarcity of adequate experimental models to investigate cone cell death. Recently, we generated *R91W;Nrl*^*−/−*^ double-mutant mice, which display a well-ordered all-cone retina with normal retinal vasculature and a strong photopic function that generates useful vision. Here we exposed *R91W;Nrl*^*−/−*^ and wild-type (*wt*) mice to toxic levels of blue light and analyzed their retinas at different time points post illumination (up to 10 days). While exposure of *wt* mice resulted in massive pyknosis in a focal region of the outer nuclear layer (ONL), the exposure of *R91W;Nrl*^*−/−*^ mice led to additional cell death detected within the inner nuclear layer. Microglia/macrophage infiltration at the site of injury was more pronounced in the all-cone retina of *R91W;Nrl*^*−/−*^ than in *wt* mice. Similarly, vascular leakage was abundant in the inner and outer retina in *R91W;Nrl*^*−/−*^ mice, whereas it was mild and restricted to the subretinal space in *wt* mice. This was accompanied by retinal swelling and the appearance of cystoid spaces in both inner and ONLs of *R91W;Nrl*^*−/−*^ mice indicating edema in affected areas. In addition, basal expression levels of tight junction protein-1 encoding ZO1 were lower in *R91W;Nrl*^*−/−*^ than in *wt* retinas. Collectively, our data suggest that exposure of *R91W;Nrl*^*−/−*^ mice to blue light not only induces cone cell death but also disrupts the inner blood–retinal barrier. Macular edema in humans is a result of diffuse capillary leakage and microaneurysms in the macular region. Blue light exposure of the *R91W;Nrl*^*−/−*^ mouse could therefore be used to study molecular events preceding edema formation in a cone-rich environment, and thus potentially help to develop treatment strategies for edema-based complications in macular degenerations.

Human vision largely depends on cone photoreceptors. As the incidence of cone degenerative diseases such as age-related macular degeneration is expected to rise in the future, the understanding of cone physiology and pathophysiology is urgently needed to develop therapeutic approaches for the preservation of cone-mediated vision in patients. Recently, we engineered an *R91W;Nrl*^*−/−*^ mouse model^1^ to analyze the impact of a human-blinding mutation found in RPE65 (the R91W) specifically on cone photoreceptors.^2, 3^ The lack of the neural retina leucine zipper (NRL) transcription factor drives all photoreceptor progenitor cells to a cone fate.^4^ Therefore, the impact of the R91W mutation on cones can be analyzed without the ‘contaminating' presence of rods in *R91W;Nrl*^*−/−*^ mice. In addition, as the *R91W* mutation leads to a hypomorphic RPE65 protein substantially reducing levels of 11-*cis*-retinal in the retina,^2, 5^ the disturbed cone layering with rosette formation typically found in the *Nrl*^*−/−*^ mouse retinas is corrected in double-mutant *R91W;Nrl*^*−/−*^ mice. Thus, the *R91W;Nrl*^*−/−*^ mouse constitutes a model with a well-ordered and functional all-cone retina.^1^

The acute model of light-induced retinal degeneration uses short exposure to bright white light to study photoreceptor cell death leading to loss of vision.^6, 7^ High photon flux, oxygen tension and the high levels of polyunsaturated fatty acids present in rod outer segment membranes make rod photoreceptor cells especially vulnerable to photochemical damage. Although light affects rod photoreceptors primarily, cones seem to be more resilient surviving for a prolonged period of time after light exposure.^8^ Cones eventually do die, but secondarily to the loss of rod cells. Endotoxins released by degenerating rods,^9^ the lack of trophic and mechanical support^10, 11^ after loss of rod cells or sudden exposure to increased oxygen levels in the absence of rods^12^ have been implicated in the secondary cone cell death.

Mammalian animal models with higher cone percentage such as gray squirrels (60% cones) or Nile rats (33% cones) showed high resistance of cones to light-induced damage.^13^ Similarly, short-term (hours) or constant (up to several months) exposure of *Nrl*^*−/−*^ mice to bright white light did not induce cone degeneration.^14, 15^ However, in the monkey retina S-cones were irreversibly damaged with high levels of monochromatic blue light.^16^ In rats and mice, high irradiances to shorter wavelengths are also more damaging to photoreceptors than broad-bandwidth light.^17^ This suggests that if conditions including exposure duration, light intensity and wavelength are appropriately chosen, the light damage model can be applied to study cone degenerations.

Here we exposed the *R91W;Nrl*^*−/−*^ mice to toxic blue light levels to induce cone cell death. We show that the all-cone retina of *R91W;Nrl*^*−/−*^ mice can be damaged, although to a lesser extent than the rod-dominant mouse retina. While blue light damage (BLD) in wild-type (*wt*) mice causes breakdown of the retinal pigment epithelium (RPE), it affects the inner blood–retinal barrier (BRB) in *R91W;Nrl*^*−/−*^ mice. Vascular leakage is accompanied by retinal swelling and edema, which seems to be more prominent in the all-cone retina.

## Results

**Figure 1:**
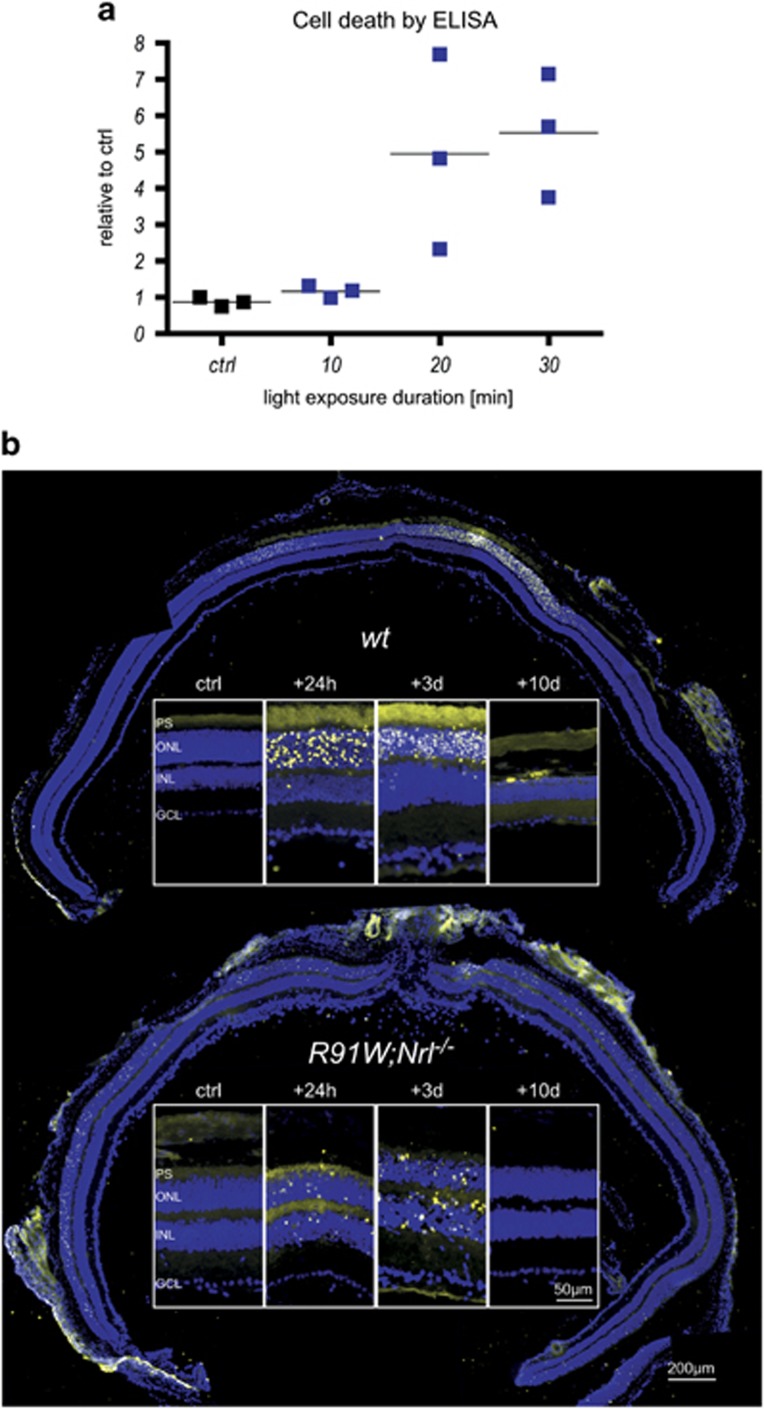
Retinal cell death after blue light exposure. (**a**) Dose–response for blue light-induced damage in the *R91W;Nrl*^*−/−*^ all-cone retina. *R91W;Nrl*^*−/−*^ mice were exposed for 10–30 min to blue light (410±10 nm; 60 mW/cm^2^) and cytoplasmic nucleosomes in the retina were quantified using an ELISA-based cell death detection kit 48 h after exposure. Horizontal lines represent mean values of *N*=3 animals. Levels of unexposed control (ctrl) retinas were set to 1. (**b**) Apoptotic cells detected by TUNEL on retinal cross-sections of unexposed control (ctrl) and exposed eyes at the time points indicated. DAPI (blue) was used to visualize cell nuclei. Retinal panoramas show representative examples at 24 h following BLD. Panels show most affected retinal areas at a higher magnification. PS: photoreceptor segments; ONL: outer nuclear layer; INL: inner nuclear layer; GCL: ganglion cell layer. *N*=3. Scale bars as indicated

To establish the blue light sensitivity of the all-cone retina we exposed *R91W;Nrl*^*−/−*^ mice to 410 nm light for up to 30 min and measured retinal cell death by ELISA 48 h after BLD (Figure 1a). As little as 2 min of exposure induced loss of photoreceptors in *wt* mice (not shown^18^). In contrast, *R91W;Nrl*^*−/−*^ mice were much more resistant to BLD and only prolonged exposure (20 and 30 min) led to cell death (Figure 1a). As the 20 min exposure led to a higher variability in damage severity, we used a 30 min exposure for all further experiments.

To localize dying cells, *R91W;Nrl*^*−/−*^ and *wt* mice were analyzed by TUNEL assay 24 h, 3 and 10 days after BLD and compared with non-exposed controls (ctrl). TUNEL-positive cells in *wt* mice were detected only in a focal area (termed hotspot^17^) in the central retina (Figure 1b). At 24 h and 3 days post exposure, almost all TUNEL-positive cells were found in the outer nuclear layer (ONL) and only occasionally in the inner nuclear layer (INL) of *wt* mice. Ten days after BLD almost all nuclei in the hotspot area were lost (Figure 1b). In *R91W;Nrl*^*−/−*^ mice, however, TUNEL-positive cells were not only found centrally but also in the periphery (Figure 1b). Dying cells were detected both in the ONL and INL at 24 h and 3 days post exposure. Overall, *R91W;Nrl*^*−/−*^ mice had fewer TUNEL-positive cells than *wt* mice, suggesting that BLD was less severe than in *wt* mice.

**Figure 2:**
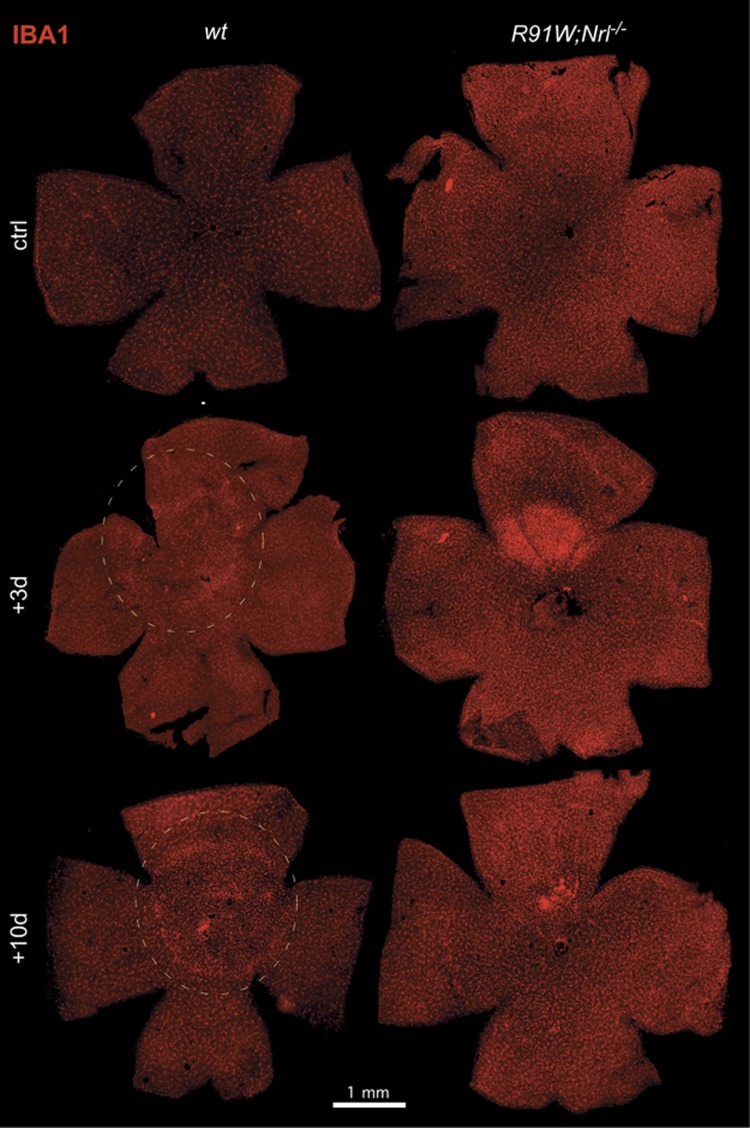
Retinal flat mounts of *wt* and *R91W;Nrl*^*−/−*^ mice before (ctrl) or at 3 and 10 days after BLD stained for IBA1, a microglia/macrophage marker. Dotted circular line marks the hotspot region in *wt*. Note that the hotspot region is visible in all light-damaged retinas, but larger regions were affected in *wt* mice. *N*=3. Scale bar as indicated

**Figure 3:**
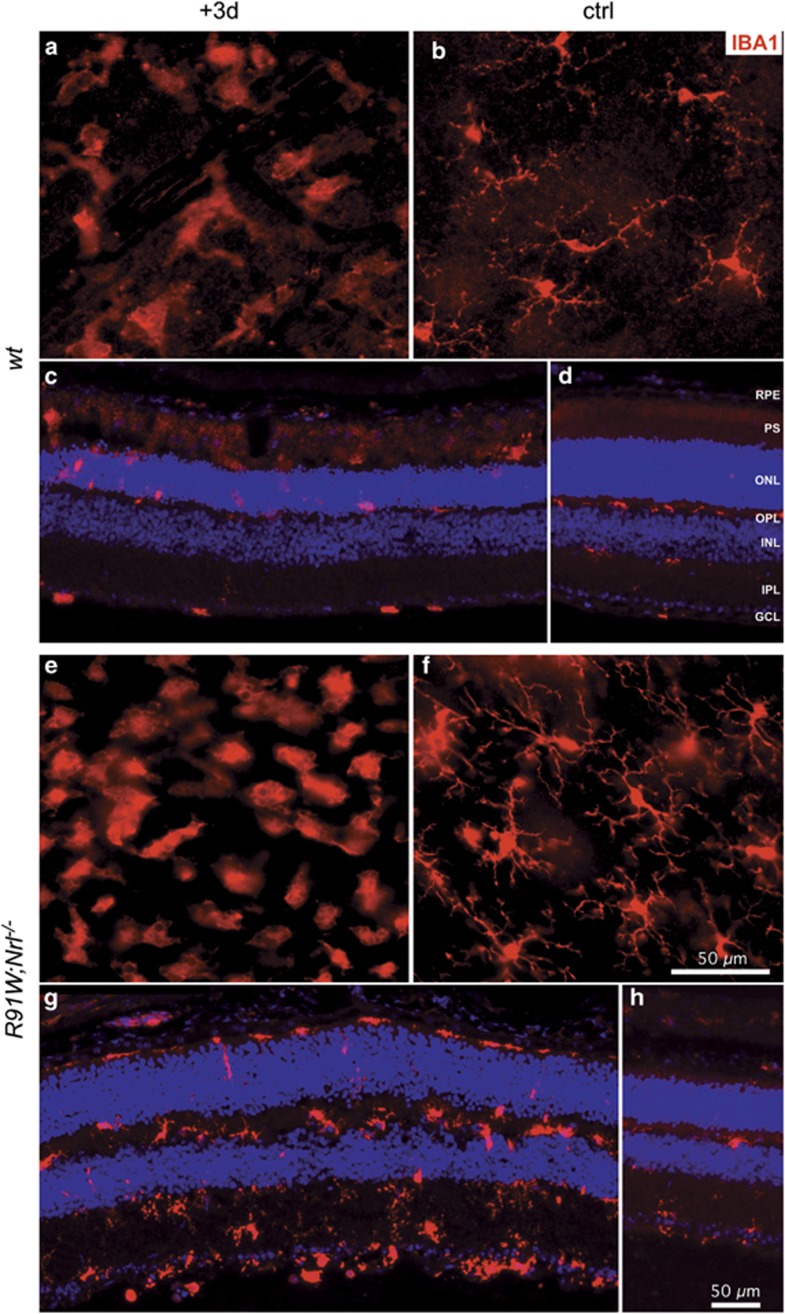
Microglia/macrophage accumulation within the hotspot area at 3 days (+3d; **a**, **c**, **e**, **g**) following BLD in *wt* and *R91W;Nrl*^*−/−*^ retinas. Undamaged regions outside the hotspot served as internal controls (ctrl; **b**, **d**, **f**, **h**). Shown are retinal flat mounts (**a**, **b**, **e**, **f**; the focal plane in the outer plexiform layer) or retinal cryosections (**c**, **d**, **g**, **h**) immunostained for IBA1. RPE: retinal pigment epithelium; OPL: outer plexiform layer; IPL: inner plexiform layer; other abbreviations as in Figure 1. *N*=3. Scale bars as indicated

BLD induces focal photoreceptor death and accumulation of microglia/macrophages in the hotspot region of *wt* mice.^18^ Therefore, we stained retinal flat mounts for IBA1, a marker for microglia and macrophages (Figures 2 and 3). In *wt* mice, density of IBA1-positive cells was clearly increased in a focal area at 3 days and remained detectable even 10 days after the light insult (Figure 2, left dotted circle). A similar IBA1-positive region was identified in *R91W;Nrl*^*−/−*^ mice. However, it was smaller in diameter and stronger in signal intensity at 3 days (Figure 2, right). Ten days after BLD, an intensely stained region was still visible in *R91W;Nrl*^*−/−*^ mice, but reduced in size suggesting disappearance of IBA1-positive cells (Figure 2, right). A closer inspection of the hotspot regions revealed accumulation of ameboid microglia in both *wt* and *R91W;Nrl*^*−/−*^ retinas at 3 days after BLD (Figures 3a and e, respectively). The number of ameboid microglia appeared higher in *R91W;Nrl*^*−/−*^, and occupied all retinal layers especially the plexiform layers as opposed to the *wt* (Figures 3c and g). Compared with the adjacent unexposed region, the hotspot area in *R91W;Nrl*^*−/−*^ seemed to be swollen with less-densely packed nuclei, especially in the INL (Figure 3, compare g with h). As expected, microglia were ramified in regions outside this central region in both mouse lines (Figures 3b, d, f and h).

**Figure 4:**
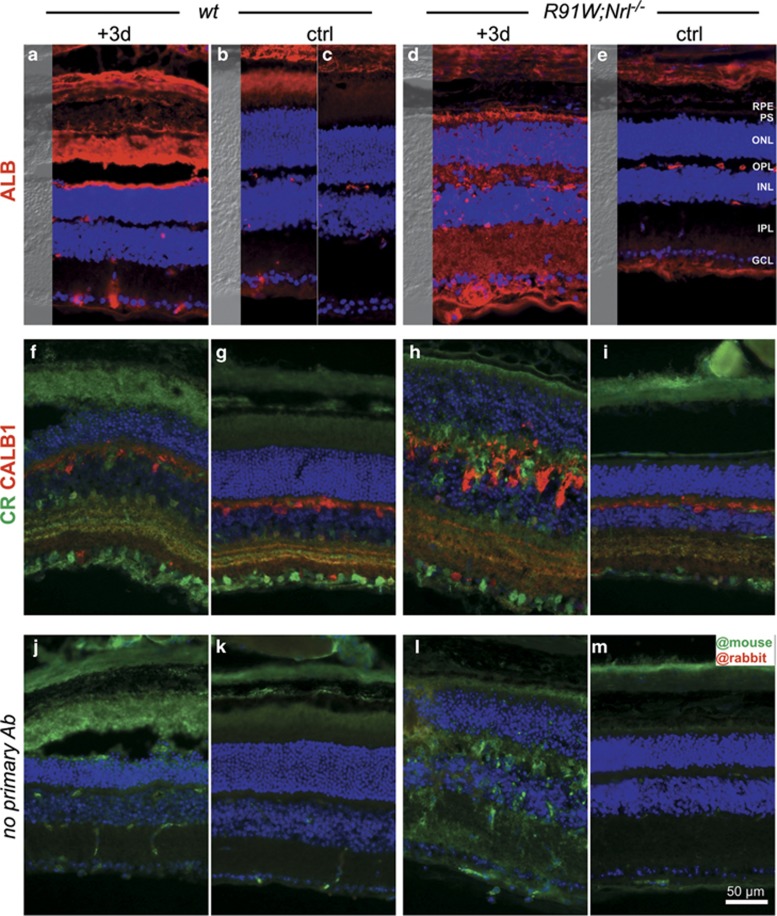
Blue light damage causes vascular leakage in the inner retina of *R91W;Nrl*^*−/−*^ mice. Mice were exposed for 30 min and analyzed 3 days following BLD. Non-damaged retinal regions outside the hotspot (ctrl; **b**, **e**, **g**, **i**, **k** and **m**) and unexposed *wt* retina (**c**) were used as controls. Staining for albumin (ALB) in *wt* (**a**–**c**) and *R91W;Nrl*^*−/−*^ (**d**, **e**) mice indicated presence of blood components. Note that ALB immunoreactivity outside the blood vessels was mostly detected in the outer retina of *wt* mice (**a**), whereas both the inner and outer retina of *R91W;Nrl*^*−/−*^ were ALB-positive (**d**). Calbindin (CALB1) and calretinin (CR) were used as markers for horizontal, amacrine and ganglion cells (**f**–**i**). CALB1 staining revealed severe disturbance of horizontal cell morphology in *R91W;Nrl*^*−/−*^ mice (**h**). Control stainings with anti-mouse secondary antibodies only showed immunoreactivity (green) in damaged (**j** and **l**) but not in neighboring undamaged retinal regions (**k** and **m**) likely due to cross-reactivity with immunoglobulins from blood that leaked to injured areas. Application of anti-rabbit secondary antibodies (**j**–**m**) did not result in a detectable signal (red). Abbreviations as in Figures 1 and 3. *N*=3. Scale bar as indicated

Blue light was shown previously to damage RPE cells thereby disrupting the outer BRB.^19^ A strong albumin staining was detected in the region between the RPE and ONL corresponding to the inner and outer photoreceptor segments (IS and OS) at 3 days post exposure in *wt* mice (Figure 4a). Albumin staining was also visible in the OS of a control region outside of the hotspot (Figure 4b). This can probably be attributed to lateral diffusion to the neighboring non-damaged area, as non-exposed *wt* retinas showed no signal in the IS and OS (Figure 4c). In contrast to *wt* mice, albumin-positive signals were found in all retinal layers in the hotspot area of *R91W;Nrl*^*−/−*^ mice (Figure 4d). This signal was specific, as the staining was restricted to the vessels of the three vascular plexi outside of the hotspot region (Figures 4e and j–m).

To test if cells within the inner retina were affected by BLD we labeled horizontal cells with anti-calbindin, and amacrine and ganglion cells with anti-calretinin antibodies, respectively. Normal distribution for both markers was found in *wt* mice (Figures 4f and g). In *R91W;Nrl*^*−/−*^ mice calbindin-positive horizontal cells were especially enlarged and displaced in the damaged region, whereas signal distribution and intensity in undamaged neighboring areas were similar to *wt* (Figures 4h and i).

Thus BLD increased vascular permeability, but affected *wt* and *R91W;Nrl*^*−/−*^ mice differently. In *wt* mice, the leakage was found predominantly in the subretinal region and probably originated from disruption of the RPE. In all-cone retinas, BLD affected the inner BRB, which was accompanied by horizontal cell disorganization.

**Figure 5:**
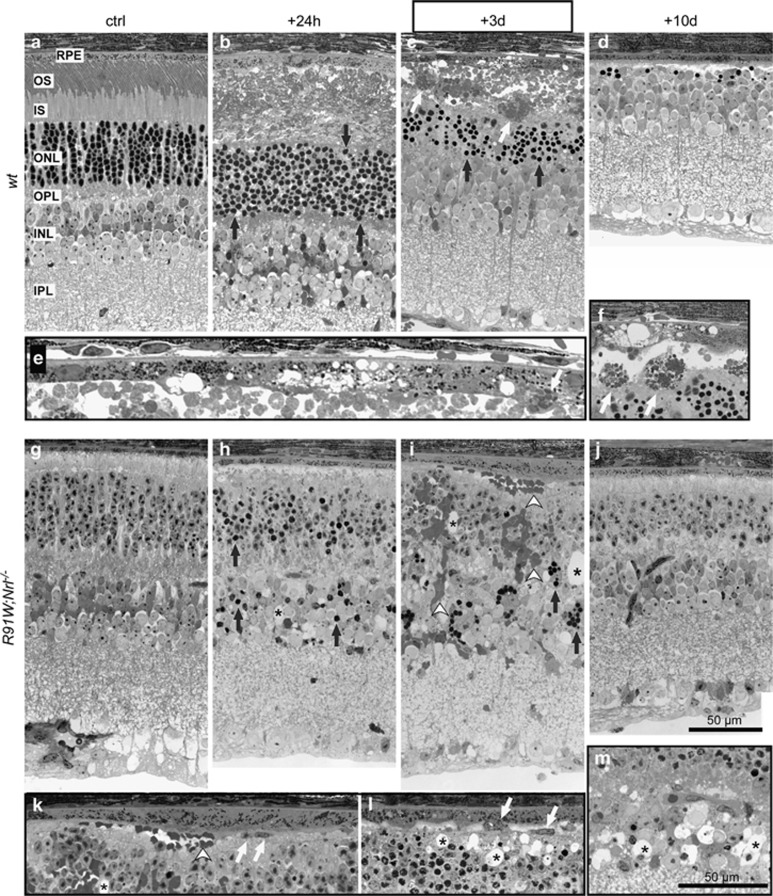
Retinal morphology of *wt* (**a**–**f**) and *R91W;Nrl*^*−/−*^ (**g**–**m**) mice at indicated time points after blue light exposure. Boxed images (**e**, **f**, **k**–**m**) are high-magnification images of retinas at the +3d time point. All photoreceptors within the hotspot were pyknotic (black arrows) in *wt* mice at 24 h (**b**). ONL thinning, subretinal accumulation of large cells, presumably microglia/macrophages (white arrows), disruption and vesiculation of the RPE was detected 3 days after the insult (**c**, **e**, **f**). No photoreceptors remained in the hotspot region of *wt* at 10 days after exposure (**d**). In *R91W;Nrl*^*−/−*^ mice pyknotic nuclei were detected in the ONL but also in the INL at 24 h after BLD (**h**). Three days following light damage (**i**, **k**–**m**) blood cells (white arrowheads) appeared in the OPL, ONL and in the subretinal space (**i**, **k**). Cystoid spaces (*) emerged throughout the outer retina (**i**, **k**, **l**) including the INL (**m**). The RPE occasionally thickened (**i**, **k**) and macrophage/microglia (white arrows) cells were detected in the subretinal space (**l**). Ten days after BLD, only a reduced number of nuclei in the ONL revealed the position of the hotspot. All other signs of blue light-induced damage have been resolved (**j**). Black arrows: pyknotic nuclei. White arrows: macrophage/microglia. Arrowheads: blood cells. *: cystoid spaces. OS: outer segments, IS: inner segments, other abbreviations as in Figures 1 and 3. Scale bars are as indicated for boxed and unboxed images. *N*=3 per time point and strain

We next analyzed retinal morphology of both strains up to 10 days after BLD. In *wt* mice, almost all photoreceptor nuclei within the hotspot region were pyknotic 24 h after BLD (Figure 5, compare a and b). Pyknosis was accompanied by vesiculation of IS and OS and swelling of the RPE (Figure 5b). RPE ruptured 3 days post exposure and large macrophages were mobilized to the region between the RPE and ONL (Figures 5c, e and f). Ten days after BLD, the RPE recovered but almost all photoreceptor nuclei were lost and no OS and IS remained in the hotspot area (Figure 5d).

In *R91W;Nrl*^*−/−*^ mice, fewer pyknotic cells were detected in the ONL 24 h after BLD (Figure 5h). Consistent with the TUNEL stainings (Figure 1b), some pyknotic nuclei were also visible in the INL (Figure 5h). Three days after BLD some retinal areas showed massive vascular leakage extending from the inner retina to the subretinal space (Figure 5i). Cystoid spaces were detected in INL, outer plexiform layer, ONL and subretinally (✶ in Figures 5h, i and k–m). In some instances blood cells were observed subretinally close to the RPE (Figures 5i and k). In the most affected regions variations in RPE thickness were visible but no obvious rupture was detected (Figures 5k and l). Larger cells, presumably activated microglia or macrophages were detected in the subretinal space (Figure 5l, white arrows). Ten days after the insult the remaining cones and RPE seemed to have recovered completely in the most affected areas of *R91W;Nrl*^*−/−*^ mice (Figure 5j).

**Figure 6:**
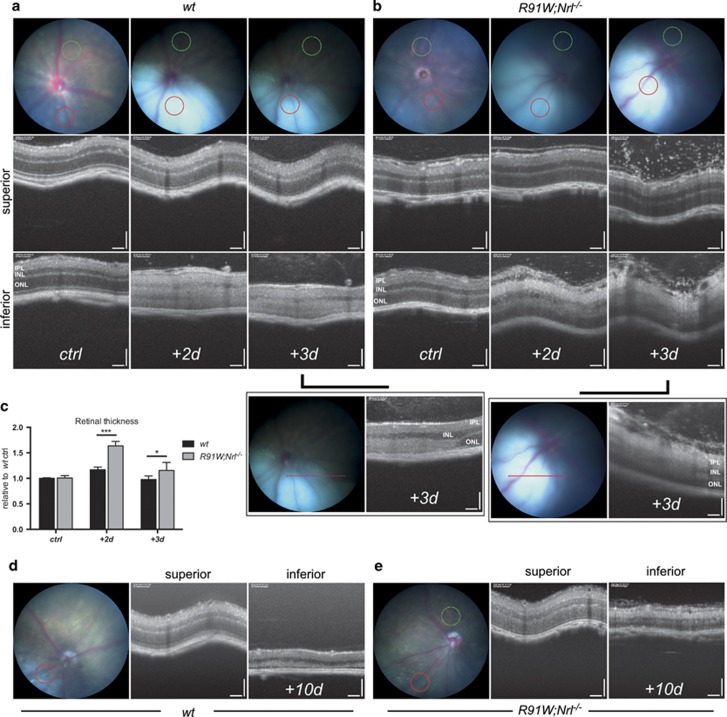
Fundus (color) and corresponding OCT (black and white) images of *wt* (**a**, **d**) and *R91W;Nrl*^*−/−*^ (**b**, **e**) mice taken up to 10 days following BLD. The positions of OCT scans are shown in fundi as colored circles/lines (green, superior; red, inferior). At 2 and 3 days after BLD the hotspot regions appeared as a pale bluish spot, much lighter than the rest of the fundus (**a**, **b**). OCT revealed that INL and ONL in the damaged (inferior) but not the control area (superior) became hyperreflective in *wt*; whereas hyperreflectivity was very pronounced in the IPL but absent in the ONL in *R91W;Nrl*^*−/−*^ mice. Boxed panels in (**a**) and (**b**) show linear scans of the transition zones analyzed 3 days following BLD. Increased retinal thickness was especially prominent in *R91W;Nrl*^*−/−*^ mice. Quantification of retinal thickness in *R91W;Nrl*^*−/−*^ and *wt* eyes that were unexposed (ctrl) or exposed to blue light, as indicated (**c**). Values are expressed relative to mean value of unexposed *wt* mice that was set to 1 (*N*=4 *wt*, 5 *R91W;Nrl*^*−/−*^; *P<0.05; ***P<0.001). At 10 days after BLD the hotspot regions can be recognized by whitish material appearing in the fundus and by a thinned retina in OCT (**d**, **e**). Note: the second and third time points in panels (**a**) and (**b**) show data from the same mouse followed for two consecutive days. Scale bars 100 *μ*m

Our data suggested an outer BRB breakdown after BLD in *wt* retinas supporting an earlier work reporting RPE disruption and retinal fluid influx after blue light exposure.^19^ This prompted us to analyze the consequences of possible fluid leakage in each model *in vivo*. Mice were examined at 2, 3 and 10 days after BLD by funduscopy and optical coherence tomography (OCT) imaging (Figure 6). Retinal funduscopy revealed a well-defined hotspot paler than the remaining retina 2 and 3 days after BLD (Figures 6a and b). This color change was previously postulated to be the result of subretinal fluid accumulation.^18^ The fundus appearance was very similar in *wt* and *R91W;Nrl*^*−/−*^ mice up to 3 days post illumination (Figures 6a and b). However, at 10 days post illumination more autofluorescent material was visible in the hotspot region in the inferior *wt* mouse retina (Figure 6d). OCT images were taken to compare the retinal structure in the superior (control) and inferior (hotspot) region of the same eye. OCT scans of the injured areas revealed an overall change in retinal reflectivity at 2 and 3 days after light exposure (Figures 6a and b). This change in reflectivity was particularly visible on linear scans of transition zones covering both the hotspot and neighboring, non-exposed area (Figures 6a and b, boxed panels). In the injured area of *wt* mice a clear distinction between the layers was lost and both nuclear layers became hyperreflective (Figure 6a). Retinas appeared swollen especially at 2 days after light damage, which was confirmed by measurement of the retinal thickness (Figure 6c). In *R91W;Nrl*^*−/−*^ mice the ONL retained its darker appearance while the INL and especially the IPL showed hyperreflectivity (Figure 6b). A strong increase in retinal thickness and loss of a clear separation between the layers within the inner retina was visible 2 and 3 days after BLD in *R91W;Nrl*^*−/−*^ mice (Figures 6b and c). In addition, a number of punctate spots were observed epiretinally (vitreal side) in both superior and inferior OCT scans especially 3 days after BLD (Figure 6b). Ten days following the light insult, OCT scans showed a clear reduction in retinal thickness in the hotspot of both mouse lines (Figures 6d and e).

**Figure 7:**
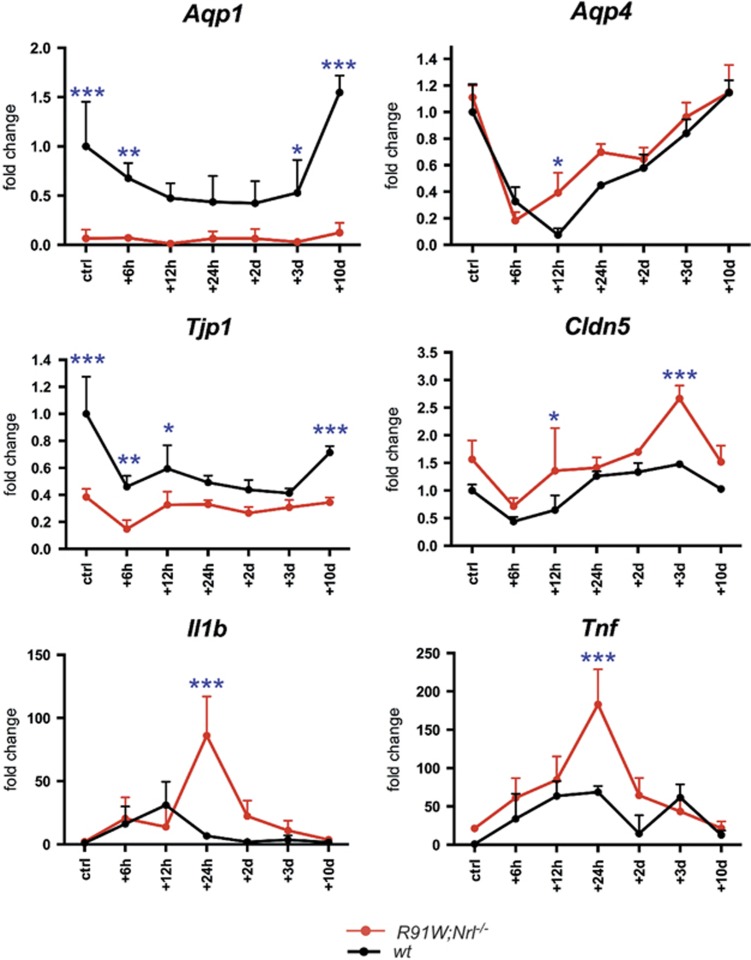
Reduced basal expression of *Aqp1* and *Tjp1* in the retina of *R91W;Nrl*^*−/−*^ all-cone mice. Expression levels of indicated genes were analyzed before (ctrl) and at six time points (as indicated) after BLD by semiquantitative real-time PCR. Expression is shown relatively to unexposed *wt* (ctrl), which was set to 1. Shown are means±S.D. *N*=3. Two-way ANOVA was used to compare expression levels between *R91W;Nrl*^*–/–*^ (red line) and *wt* (black line) mice at individual time points. *P<0.05; **P<0.01; ***P<0.001. *Aqp,* aquaporin; *Tjp*, tight junction protein; *Cldn*, claudin; *Il1b*, interleukin 1 beta; *Tnf*, tumor necrosis factor

OCT and morphological analysis revealed edema formation in both mouse models. This prompted us to analyze the gene expression levels of aquaporins (*Aqp*) and tight junction proteins as they are important for maintaining the retinal fluid balance and the integrity of the BRB. Alterations in AQP1 and AQP4 expression have previously been detected in a rat model of BLD.^20^ Decreased retinal expression of *Aqp1* was found in *wt* mice during the first 3 days following BLD. After that, expression recovered and reached or even surpassed control levels at 10 days after exposure (Figure 7). Surprisingly, retinas of *R91W;Nrl*^*−/−*^ mice showed very low expression levels of *Aqp1* at all time points tested (Figure 7). As strong *Aqp1* expression was previously found in photoreceptors^21^ our data suggest that *Aqp1* is mainly expressed by rods but not by cones. A similar pattern of expression characterized by an initial sharp decrease following the light insult was observed for *Aqp4* in both mouse strains. *Wt* mice had a slightly delayed recovery of expression as judged by the 12 h time point (Figure 7). Claudins, including CLDN5, compose the major structural and functional elements of tight junctions between endothelial cells forming the inner BRB. In both mouse lines BLD caused an initial drop in transcription (6 h time point). *Cldn5* expression levels recovered fast and were stabilized at 24 h in *wt,* whereas in *R91W;Nrl*^*−/−*^ mice they peaked at 3 days after BLD. The expression of tight junction protein-1 (*Tjp1)* dropped in the *wt* and *R91W;Nrl*^*−/−*^ mice 6 h after BLD. Surprisingly, *Tjp1* levels were low in *R91W;Nrl*^*−/−*^ mice in general. In addition, the expression of the proinflammatory cytokines *Il1b* and *Tnf* was highly increased in *R91W;Nrl*^*−/−*^, especially 24 h after BLD (Figure 7). Both cytokines were also upregulated in *wt* mice after BLD insult, but to a lesser extent (Figure 7).

Collectively, our data suggest low basal expression of *Aqp1* and *Tjp1* in *R91W;Nrl*^*−/−*^mice. Differential expression of these genes and thus alterations in water homeostasis and tight junction formation in retinas of the all-cone *R91W;Nrl*^*−/−*^mice may account for the strong edema formation observed in *R91W;Nrl*^*−/−*^ mice after BLD.

## Discussion

The aim of this study was to analyze light-induced retinal degeneration in the all-cone *R91W;Nrl*^*−/−*^ mouse and compare it to the rod-dominant *wt* mouse. By using the *R91W;Nrl*^*−/−*^ mouse model, we directly showed that blue light could induce cone cell death. However, cone photoreceptors were more resistant to BLD than rods. Degeneration was accompanied by the appearance of microglial cells in the injured area. Although activated microglia were mostly restricted to the damaged outer retina in *wt* mice, microglial cells were detected in all layers of the damaged *R91W;Nrl*^*−/−*^ retina. Degeneration was accompanied by a more pronounced edema formation in *R91W;Nrl*^*−/−*^ mice, and a strongly reduced basal expression of *Aqp1* and *Tjp1* that are involved in water extrusion and the formation of tight junctions, respectively. Also, an increased expression of *Tnf* and *Il1b* further corroborates a stronger local inflammatory response and edema in *R91W;Nrl*^*−/−*^ mice after BLD.

Photoreceptor damage after blue light exposure was more pronounced in rod-dominant *wt* retinas with almost all photoreceptors lost within the hotspot in *wt* mice (Figure 5). This was accompanied by impairment of RPE integrity 3 days after light exposure. Although RPE changes were observed also in *R91W;Nrl*^*−/−*^ mice, vesiculations and RPE rupture were only detected in exposed *wt* retinas. In theory, RPE cells of *R91W;Nrl*^*−/−*^ mice should receive much more light due to the reduced levels of visual pigments, which are the main light absorbers in the retina. This argues that the RPE rupture observed in *wt* mice was not a result of direct photon-damage (photostress) to the RPE cells, but that it may be a secondary event. Organisciak and colleagues^7^ hypothesized that the removal of light-damaged outer segments by the phagocytic activity of RPE cells may be responsible for oxidative stress and consequent RPE injury. In other words, RPE cells of *wt* mice may be poisoned by a surplus of toxic phagocytic material. In addition, an excess of all-trans-retinal diffusing from damaged photoreceptors could also be toxic for RPE cells.^22^ As the photoreceptor damage in *R91W;Nrl*^*−/−*^ is not as strong and photoreceptor segments are shorter with less-chromophore present, phagocytosis of the debris after BLD may be less challenging for the RPE, and the potential toxicity of all-*trans*-retinal may be reduced in the all-cone retina. Alternatively, basic antioxidative defense mechanisms might be enhanced in the RPE of *R91W;Nrl*^*−/−*^ mice as an adaptation to the potentially increased light levels reaching the RPE. This may render RPE cells in *R91W;Nrl*^*−/−*^ mice more resistant, a hypothesis that will be tested in future experiments.

Shorter outer segments and lower chromophore levels reduce the maximal photon catch capacity of cones leading to a reduced light absorption in retinas of *R91W;Nrl*^*−/−*^ mice in general. As a consequence, incoming light is not efficiently absorbed by photoreceptors and photons are thus more likely to scatter in the retina. Such an increased intraretinal light scattering in *R91W;Nrl*^*−/−*^ mice may explain the increased number of TUNEL-positive cells in the retinal periphery of these mice (Figure 1) as well as the damage in the inner retina where we detected vascular leakage, appearance of cystoid spaces, retinal swelling and horizontal cell hypertrophy. Although it is unclear, which cells were damaged in the INL, we speculate that some Müller glia cells might have been affected. Indeed, it was recently shown that ablation of Müller cells caused partial breakdown of the inner BRB leading to vascular leakage,^23^ a phenomenon resembling our observations presented here (see below). We cannot exclude, however, that also some interneurons were affected but as no gross changes in INL thickness and morphology were detected at late time points (10 days, Figure 5j), BLD only mildly affected cells of the INL.

Albumin staining revealed that BLD caused an outer BRB breakdown in *wt* mice (Figure 4). The resulting edema was especially prominent 2 days post exposure as evidenced by OCT analysis (Figure 6). Development of local edema within the outer retina after excessive light exposure has been described in the literature as a consequence of RPE damage as well as of the normotonic shrinkage of cells undergoing apoptosis.^24^ Although the outer BRB was compromised in *wt* mice, the inner BRB was affected in addition in *R91W;Nrl*^*−/−*^ mice: intraretinal vascular leakage and albumin immunoreactivity was detected in all retinal layers (Figures 4 and 5). Furthermore, erythrocytes were found in the subretinal space next to a thickened but not ruptured RPE (Figure 5i). Although data suggest a disruption of the inner BRB, it is still possible that tight junctions in the RPE were affected loosening cell–cell contacts and contributing to edema formation in the all-cone retinas of *R91W;Nrl*^*−/−*^ mice after BLD. Retinal hemorrhages can cause serious problems and vision loss in human patients. Yet, little is known which direct cytotoxic consequences the extravasated blood components have on cells and tissues. Protoporphyrin IX, a blood-borne photosensitizer, was shown to produce free radicals that can damage cells.^25^ An early work in a rabbit model of experimental subretinal hemorrhages suggested hemoglobin toxicity^26^ that was further substantiated in various models showing that cell-free hemoglobin and iron are strong neurotoxins.^27^ Thus, it seems likely that apoptosis detected in the INL of *R91W;Nrl*^*−/−*^ mice was not a direct consequence of light exposure but was indirectly caused by toxic effects of extravasated blood components. A recent report attributed photoreceptor degeneration in a mouse model of subretinal hemorrhage to the presence of blood constituents in the tissue and demonstrated the involvement of an inflammatory reaction in governing the severity of degeneration.^28^ In our model, large macrophages and activated microglia were mobilized to the outer retina in *wt* mice. In *R91W;Nrl*^*−/−*^ mice, however, these immune cells were increased in numbers and not only restricted to the outer retina but found in all layers. This was accompanied by a significantly stronger induction of *Tnf* and *Il1b* expression in *R91W;Nrl*^*−/−*^ than in *wt* mice (Figure 7). It is important to note that microglial activation impairs BBB function by the release of various proinflammatory factors including TNF and IL1*β*, leading to a hyperpermeability shown to be associated with neurodegenerative disorders such as Alzheimers disease and multiple sclerosis (reviewed in ref. 29).

We detected BLD-induced edema formation in both mouse models by fundus imaging and OCT (Figure 6). However, swelling was not only more pronounced in *R91W;Nrl*^*−/−*^ but it also persisted for a longer period than in *wt* mice (Figures 6a–c). The integrity of blood vessels and the regulation of water flux are important for the BRB and the maintenance of a physiologic tissue environment, respectively. Barrier function depends on tight junction proteins such as TJP1 (ZO1) in endothelial cells and water flux in the outer retina is at least partly regulated through AQP1 channels residing in the RPE and photoreceptor cells.^30^ Reduced expression of *Tjp1* increases barrier permeability^31^ and downregulation of *Aqp1*, among other factors, has been proposed to contribute to edema development in a rat model of branch retinal vein occlusion.^32^ As *R91W;Nrl*^*−/−*^ mice had significantly reduced basal expression levels of both, *Tjp1* and *Aqp1* in the retina (Figure 7), they may not only be prone to increased vascular leakage upon stress, but may also clear accumulated liquid less efficiently from the retinal tissue leading to prolonged edema.

In summary, we have analyzed the consequence of BLD for the all-cone retina. We show substantial cone degeneration after BLD that is accompanied with vascular leakage and strong edema formation. BLD *R91W;Nrl*^*−/−*^ mice can be used as a platform to test potential therapeutic agents to prevent osmotic swelling and impaired fluid absorption, which, if left untreated in patients, may result in a severe visual impairment and blindness. Pharmacologic stimulation of purinergic receptor signaling may be a promising direction of research^33^ and has already shown efficacy in a brain injury model.^34^

## Materials and Methods

### Mice

All animal experimentation adhered to the ARVO Statement for the Use of Animals in Ophthalmic and Vision Research and the regulations of the Veterinary Authorities of Kanton Zurich, Switzerland. The *R91W;Nrl*^*−/−*^ strain was described recently.^1^ 129S6 wild-type mice (Taconic, Ejby, Denmark) served as controls. Both mouse lines were housed in the animal facility of the University of Zurich in a 12 h:12 h light–dark cycle with access to food and water *ad libitum.* At the time of experimentation, the mice were between 6 and 8 weeks of age.

### Blue light exposure and quantification of retinal damage

Blue light exposure was described recently in detail.^35^ In brief, mice were dark-adapted overnight, pupils were dilated with cyclogyl 1% (Alcon Pharmaceuticals, Fribourg, Switzerland) and phenylephrine 5% (Ursapharm, Saarbrücken, Germany) in dim red light. Approximately 5 min before exposure, the mice were anesthetized subcutaneously with ketamine (85 mg/kg; Inresa Arzneimittel, Freiburg, Germany) and xylazine (4 mg/kg Bayer AG, Leverkusen, Germany) and placed on a pre-warmed surface. To keep both eyes moist during exposure, 2% methocel (OmniVision AG, Neuhausen, Switzerland) was applied. The left eyes were exposed for 10–30 min to blue light (410±10 nm; 60 mW/cm^2^ at the level of the cornea). Unexposed eyes served as controls. Following light exposure mice were kept in darkness overnight; and then returned to the normal light/dark cycle until analysis.

The extent of light-induced damage was assessed in retinas of *R91W;Nrl*^*−/−*^ mice exposed for 10, 20 and 30 min to blue light. Forty-eight hours after light exposure, apoptotic cell death was quantified in isolated retinas using the ELISA-based determination of free nucleosomes in the cytoplasm (Cell Death Detection ElisaPlus, 1920685; Roche Diagnostics, Basel, Switzerland) according to the manufacturer's recommendation.

### Immunofluorescence and retinal flat mounts

Mice were euthanized and eyes were marked dorsally by cauterization, enucleated, fixed in 4% PFA and processed for cryosectioning, as described earlier.^36^ Naso-temporal cryosections (12 *μ*m) were blocked for 1 h with 3% normal goat serum (containing 0.3% Triton X-100 in PBS), and incubated overnight at 4 °C with the following primary antibodies: rabbit anti-albumin (ALB) (1 : 500, RARaAlb; Nordic Immunology, Tilburg, Netherlands), rabbit anti-calbindin (CALB) (1 : 500, AB1778; Chemicon, Temecula, CA, USA), mouse anti-calretinin (CR) (1 : 1000, AB5054; Chemicon), and rabbit anti-allograft inflammatory factor 1 (alias IBA1) (1 : 1000, 019-19741; Wako, Neuss, Germany). After washing, slides were incubated with appropriate secondary antibodies labeled with Cy2 or Cy3 (Jackson ImmunoResearch Laboratories, Soham, UK), counterstained with 4′,6-diamidino-2-phenylindole (DAPI; Life Technologies, Zug, Switzerland), and analyzed with a digitalized microscope (Zeiss Axioplan, Jena, Germany).

For preparation of retinal flat mounts enucleated eyes were incubated for 20–30 min in 2% PFA prepared in PBS. Cornea and lens were removed and the retina was dissected from the sclera and flat-mounted in PBS. Retinal flat mounts were postfixed in 4% PFA for 10 min, washed in PBS, and blocked with 3% normal goat serum for 1 h. Flat mounts were incubated with anti-IBA1 (1 : 500, Wako) overnight. After washing in PBS, flat mounts were incubated with Cy3-labeled secondary antibody (Jackson ImmunoResearch Laboratories), washed, mounted and immunofluorescence staining was analyzed with microscope (Zeiss Axioplan).

### *In situ* cell death detection (TUNEL)

For TUNEL assay air-dried cryosections were postfixed for 10 min with 4% PFA, washed for 10 min in PBS before the tissue was permeabilized with freshly prepared 0.1% Triton X-100 in 0.1% sodium citrate for 3 min. The slides were washed in PBS twice for 1 min and labeled with the *In Situ* Cell Death Detection Kit (Roche Diagnostics, Rotkreuz, Switzerland, Cat. No. 11 684 795 910). Five *μ*l terminal deoxynucleotidyl transferase enzyme solution and 45 *μ*l label solution, containing fluorescein labeled deoxyuridine-triphosphate, were mixed and applied to the slides and incubated for 1 h at 37 °C in a humid chamber. Slides were washed, counterstained with DAPI (Life Technologies), mounted and analyzed as described above.

### Morphology

The detailed procedure was described recently.^36^ In brief, eyes were marked dorsally, enucleated and fixed in 2.5% glutaraldehyde. Dorsal and ventral eye halves were separated by a cut through the optic nerve head and separately embedded in epon plastic. For light microscopy, semithin cross-sections (0.5 *μ*m) were counterstained with toluidine blue and analyzed with a digitalized microscope (Zeiss Axioplan).

### Fundus imaging and OCT

Five minutes prior to fundus imaging, the pupils were dilated and mice were anesthetized as described above. During imaging, the eyes were lubricated with methocel 2% (OmniVision AG). Retinal fundus images were taken during the same session as corresponding OCT scans using the MicronIV fundus camera and OCT Scan Head equipped with the mouse objective nosepiece with integrated polarizing beam splitter (Phoenix Research Labs, Pleasanton, CA, USA). The MicronIV was equipped with a Xenon lamp as a light source and Semrock FF01-554/211 filter limiting the wavelength range for bright filed imaging from 450 to 680 nm. The OCT device featured a broadband superluminescent diode at 830 nm customized for retinal imaging of mice. The scan region on the mouse retina was 1.8 mm in the X and the Y direction. Linear or circle OCT scans consisted of a series of 1024 single point A-Scans. OCT circle scans started at the 9 : 00 position and traveled counterclockwise. Scans from superior and inferior (damaged) retinal areas were taken and compared among the strains and conditions. Images were captured using StreamPix 5 and Micron OCT commercial softwares (Phoenix Research Labs). The two fundus images showing the upper (green circle) and lower (red circle) scan position were superimposed using Adobe Photoshop CS3 (Adobe Systems, Inc., San Jose, CA, USA). At least three mice per condition and strain were analyzed. Scale bars 100 *μ*m. Average retinal thickness, representing the distance between nerve fiber layer and the RPE (apical side), was determined using the InSight—Animal OCT Segmentation Software (Phoenix Research Labs). Retinal thickness was measured in the most affected area of the hotspot and in the unexposed contralateral eye that served as control (ctrl). Numerical data were exported for statistical analysis described below.

### RNA isolation and semiquantitative real-time PCR

Retinas were removed trough a slit in the cornea and snap-frozen in liquid nitrogen. Total retinal RNA was isolated using an RNA isolation kit (RNeasy, Qiagen, Hilden, Germany), which included a DNase treatment. One microgram of total RNA was used for reverse transcription using oligo(dT) and M-MLV reverse transcriptase (Promega, Dübendorf, Switzerland). Gene expression was analyzed by real-time PCR on 10 ng template cDNA using PCR polymerase ready mix (LightCycler480 SYBR Green I Master; Roche Diagnostics), and a LightCycler480 thermocycler (Roche Diagnostics). Specific primer pairs (Table 1) were designed to span a large intronic region or an exon/exon boundary of the target genes. Signals were normalized to *Actb* and relative expression was calculated using LightCycler480 software (Roche Diagnostics) using a calibrator sample.

### Statistical analysis

Statistical analysis was performed using Prism4 software (GraphPad, San Diego, CA, USA). All data are presented as mean values±S.D.; *N*≥3. Two-way ANOVA followed by a Bonferroni's *post hoc* test was used to determine significance. *P*-values below 0.05 were considered significant.

## Figures and Tables

**Table 1:** Primers used for semiquantitative real-time PCR

**Gene**	**Forward**	**Reverse**	**Size (bp)**
*Actb*	*CAACGGCTCCGGCATGTGC*	*CTCTTGCTCTGGGCCTCG*	*153*
*Aqp1*	*CACTTGGCCGCAATGACCT*	*CCAGAACGCACAGTACCAGC*	*96*
*Aqp4*	*TACTGGAGCCAGCATGAATC*	*CCACATCAGGACAGAAGACA*	*149*
*Tjp1*	*CAGCAGCTAAGGAAGAGTGG*	*ATTATCAGACACCGGCTCAG*	*105*
*Cldn5*	*TGCCTTCCTGGACCACAA*	*GCGCCAGCACAGATTCATA*	*119*
*Il1b*	*GCTATGGCAACTGTTCCTGA*	*GATGTGCTGCTGCGAGATT*	*171*
*Tnf*	*CCACGCTCTTCTGTCTACTGA*	*GGCCATAGAACTGATGAGAGG*	*95*

## Abbreviations

ALB: albumin
Aqp: aquaporin
BLD: blue light damage
BRB: blood–retinal barrier
CALB1: calbindin
Cldn: claudin
CR: calretinin
GCL: ganglion cell layer
IBA1: ionized calcium-binding adaptor molecule 1
Il1b: interleukin 1 beta
INL: inner nuclear layer
IPL: inner plexiform layer
IS: inner segments
NRL: neural retina leucine zipper
OCT: optical coherence tomography
ONL: outer nuclear layer
OPL: outer plexiform layer
OS: outer segments
PS: photoreceptor segments
RPE: retinal pigment epithelium
Tjp: tight junction protein
Tnf: tumor necrosis factor
*wt*: wild-type
